# Cerebellar transcranial direct current stimulation in spinocerebellar ataxia type 3 (SCA3-tDCS): rationale and protocol of a randomized, double-blind, sham-controlled study

**DOI:** 10.1186/s12883-019-1379-2

**Published:** 2019-07-04

**Authors:** Roderick P. P. W. M. Maas, Ivan Toni, Jonne Doorduin, Thomas Klockgether, Dennis J. L. G. Schutter, Bart P. C. van de Warrenburg

**Affiliations:** 1 Department of Neurology, Donders Institute for Brain, Cognition, and Behaviour, Radboud University Medical Center, Reinier Postlaan 4, 6525 GC Nijmegen, The Netherlands; 2 Donders Institute for Brain, Cognition, and Behaviour, Radboud University, Nijmegen, The Netherlands; 3 Department of Neurology, University of Bonn, Bonn, Germany; 4 German Center for Neurodegenerative Diseases (DZNE), Bonn, Germany

**Keywords:** Spinocerebellar ataxia, Transcranial direct current stimulation, Randomized controlled trial, Treatment

## Abstract

**Background:**

Spinocerebellar ataxia type 3 (SCA3) is the most common subtype among the autosomal dominant cerebellar ataxias, a group of neurodegenerative disorders for which currently no disease-specific therapy is available. Evidence-based options for symptomatic treatment of ataxia are also limited. Recent investigations in a heterogeneous group of hereditary and acquired ataxias showed promising, prolonged effects of a two-week course with daily sessions of cerebellar anodal transcranial direct current stimulation (tDCS) on ataxia severity, gait speed, and upper limb dexterity. The aim of the SCA3-tDCS study is to further examine whether tDCS improves ataxia severity and various (cerebellar) non-motor symptoms in a homogeneous cohort of SCA3 patients and to explore the time course of these effects.

**Methods/design:**

An investigator-initiated, double-blind, randomized, sham-controlled, single-center trial will be conducted. Twenty mildly to moderately affected SCA3 patients (Scale for the Assessment and Rating of Ataxia score between 3 and 20) will be included and randomly assigned in a 1:1 ratio to either cerebellar anodal tDCS or sham cerebellar tDCS. Patients, investigators, and outcome assessors are unaware of treatment allocation. Cerebellar tDCS (20 min, 2 mA, ramp-up and down periods of 30 s each) will be delivered over ten sessions, distributed in two groups of five consecutive days with a two-day break in between. Outcomes are assessed after a single session of tDCS, after the tenth stimulation (T1), and after three, six, and twelve months. The primary outcome measure is the absolute change of the SARA score between baseline and T1. In addition, effects on a variety of other motor and neuropsychological functions in which the cerebellum is known to be involved will be evaluated using quantitative motor tests, static posturography, neurophysiological measurements, cognitive assessment, and questionnaires.

**Discussion:**

The results of this study will inform us whether repeated sessions of cerebellar anodal tDCS benefit SCA3 patients and whether this form of non-invasive stimulation might be a novel therapeutic approach to consider in a neurorehabilitation setting. Combined with two earlier controlled trials, a positive effect of the SCA3-tDCS study will encourage implementation of this intervention and stimulate further research in other SCAs and heredodegenerative ataxias.

**Trial registration:**

NL7321, registered October 8, 2018.

## Background

Spinocerebellar ataxia type 3 (SCA3) is the most common subtype among the autosomal dominant cerebellar ataxias (ADCAs), a clinically and genetically heterogeneous group of progressive neurodegenerative disorders [1–3]. Ataxia has been shown to be the primary and shared driver of both motor impairment and reduced quality of life in various SCAs [4, 5]. In addition to deterioration in the motor domain, specific cognitive abilities tend to decline and depressive symptoms frequently arise during disease progression [6–13].

As therapies that specifically target the underlying molecular and cellular processes are currently not available, SCA3 remains an untreatable condition and clinical management focuses on trying to provide symptomatic relief. Unfortunately, evidence-based options for symptomatic treatment of ataxia are still limited [14]. According to the recently published recommendations by the American Academy of Neurology [15], the only drug with class I study evidence of benefit in terms of improving ataxia in SCA patients is riluzole, although it should be noted that this particular trial did not include SCA3 patients [16]. In daily practice, training, exercise, and other rehabilitation programs compose the main pillars of intervention. These strategies might be beneficial, but previous research has shown that referrals to these facilities are inconsistent, ataxia-specific expertise in allied healthcare is limited, and guidelines are lacking [17]. Apart from the requirement to improve the organization of neurorehabilitation care, there is an urgent medical need to find alternative – or synergistic – ways to provide symptomatic relief for ataxia patients.

Cerebellar transcranial direct current stimulation (tDCS) is an increasingly used, safe, cheap, and non-invasive tool in neuroscience that is able to modulate cerebellar excitability [18, 19]. Computational modeling studies have demonstrated the biophysical feasibility of modulating cerebellar structures using tDCS with only negligible spreading effects to neighboring regions [20, 21]. Initially, this technique has been successfully applied in healthy volunteers to investigate the neural correlates of motor learning and cognitive and emotional processes [19, 22, 23]. More recently, studies also started to explore its therapeutic potential in a variety of neurological conditions [24, 25]. The first promising results of this technique in ataxia patients – significant reduction of upper limb postural and action tremor and dysmetria – have been described in single cases of SCA2 and ARCA3 due to *ANO10* gene mutations [26, 27]. These effects were subsequently corroborated on a larger scale by Benussi and colleagues in a randomized, double-blind, sham-controlled study enrolling a rather heterogeneous group of nineteen patients with both hereditary and acquired ataxias. Compared to the sham condition, a single session of anodal cerebellar tDCS resulted in a significant transient improvement in scores on the Scale for the Assessment and Rating of Ataxia (SARA), International Cooperative Ataxia Rating Scale (ICARS), 9-hole peg test (9HPT), and 8 m walk test (8MWT) [28]. Furthermore, a double-blind, randomized, sham-controlled trial by the same authors assessing the efficacy of tDCS five days per week for two consecutive weeks in a heterogeneous group of twenty ataxia patients – five SCA2, one SCA14, two SCA38, one Friedreich ataxia, one ataxia with oculomotor apraxia type 2, four multiple system atrophy cerebellar type, one fragile X-associated tremor/ataxia syndrome, and five sporadic adult-onset ataxia of unknown etiology – showed a significant improvement on all of the abovementioned scores up to three months after stimulation. This clinical amelioration was paralleled by restoration of the physiological inhibition of the pathways between the cerebellum and the contralateral motor cortex, as measured with double-coil transcranial magnetic stimulation (TMS) [29]. More specifically, this study was able to achieve a SARA score reduction of almost three points up to three months after stimulation, which would nearly equal the progression in SCA3 over two years [30]. Recently, these authors published the results of another randomized, double-blind, sham-controlled trial in twenty ataxia patients with mixed etiologies and found similar results regarding symptomatic ataxia improvement and return of cerebellar brain inhibition after ten sessions of cerebello-spinal tDCS [31]. No serious adverse events of cerebellar tDCS have been reported in these and other studies and it is therefore considered a safe and tolerable method. Possible side effects mainly include a transient itching, tingling, or mild burning sensation underneath the electrodes.

Inspired by these promising findings, we designed the clinically-oriented SCA3-tDCS study to investigate whether cerebellar anodal tDCS, compared to sham stimulation, decreases ataxia severity and a variety of (cerebellar) non-motor symptoms in a homogeneous cohort of SCA3 patients and if so, what the duration of this beneficial effect is.

## Methods/design

### Study design

The SCA3-tDCS study is an investigator-initiated, double-blind, randomized (1:1), sham-controlled, single-center trial that will take place at the Radboud University Medical Center, Nijmegen, the Netherlands. Cerebellar tDCS will be delivered during five consecutive days for two weeks, with a two-day break in between. Primary and secondary outcome measures (see Tables 1 and 2) are assessed at baseline (T0 before tDCS), after a single session of tDCS (T0 after tDCS), after the tenth session of tDCS (T1), and after three, six, and twelve months of follow-up (T2, T3, and T4, respectively). For this purpose, we will use a combined approach of a standardized neurological examination, quantitative motor tests, cognitive assessment, questionnaires, and neurophysiological measurements (Table 1). This combination of tests reflects the broad range of motor, cognitive, and affective functions the cerebellum is known to be involved in, and represents recognized domains of deficit in patients with cerebellar diseases.

**Table 1:** Overview of the questionnaires, neurological tests, and kinetic and neurophysiological measurements at the various points in time of the SCA3-tDCS study

	T0 baseline	T0 after tDCS	T1 day 12	T2 3 months	T3 6 months	T4 12 months
Questionnaires						
EQ-5D-5L	X		X	X	X	X
PHQ-9	X		X	X	X	X
POMS 32-item	X		X	X	X	X
iMCQ	X					X
IPAQ parts 1 and 4	X			X		X
FARS part II (ADL)	X		X	X	X	X
Neurological examination						
CCAS scale	X		X	X	X	X
SARA	X	X	X	X	X	X
8MWT	X	X	X	X	X	X
9HPT	X	X	X	X	X	X
PATA repetition	X	X	X	X	X	X
INAS	X		X	X	X	X
Measurements						
TMS	X		X			
Delay EBC	X		X			
Static posturography	X	X	X			

*EQ-5D-5L* EuroQol 5-Dimension 5-Level, *PHQ-9* Patient Health Questionnaire-9, *POMS* Profile of Mood States, *iMCQ* Institute for Medical Technology Assessment (iMTA) Medical Consumption Questionnaire, *IPAQ* International Physical Activity Questionnaire; *FARS* Friedreich Ataxia Rating Scale, *ADL* Activities of Daily Living, *CCAS* Cerebellar Cognitive Affective Syndrome, *SARA* Scale for the Assessment and Rating of Ataxia, *8MWT* 8 m walk test, *9HPT* 9-hole peg test, *INAS* Inventory of Non-Ataxia Signs, *TMS* Transcranial magnetic stimulation, *EBC* Eyeblink conditioning

**Table 2:** Outcome measures and tests

Outcome domain	Test	Time point
Primary outcome measure		
Cerebellar ataxia severity	SARA (absolute change from baseline)	T1
Secondary outcome measures		
Cerebellar ataxia severity	SARA (% of patients with a decrease of ≥ 1.5 points)	T1
Cerebellar ataxia severity	SARA score and subscores	T0 after tDCS, T2-T4
Gait speed	8 m walk test	T0 after tDCS, T1-T4
Articulation speed	PATA repetition rate	T0 after tDCS, T1-T4
Upper limb dexterity	9-hole peg test	T0 after tDCS, T1-T4
Extracerebellar signs	INAS count	T1-T4
Postural sway	Static posturography	T0 after tDCS and T1
Quality of life	EQ-5D-5L	T1-T4
Depression	PHQ-9	T1-T4
Mood states	POMS 32-item version	T1-T4
Cognition and affect	CCAS scale (total score and number of ‘fails’)	T1-T4
Activities of daily living	FARS part II	T1-T4
Medical consumption	iMCQ	T4
Motor learning	Delay EBC (acquisition of CRs)	T1
CBI	Transcranial magnetic stimulation	T1
tDCS-related side effects	Open question	T1
Correct randomization	Patients’ thoughts about treatment allocation	T1

*SARA* Scale for the Assessment and Rating of Ataxia, *INAS* Inventory of Non-Ataxia Signs, *EQ-5D-5L * EuroQol 5-Dimension 5-Level, *PHQ-9* Patient Health Questionnaire-9, *POMS* Profile of Mood States, *CCAS* Cerebellar Cognitive Affective Syndrome, *FARS* Friedreich Ataxia Rating Scale, i*MCQ* Institute for Medical Technology Assessment (iMTA) Medical Consumption Questionnaire, *EBC* Eyeblink conditioning; *CR* Conditioned response, *CBI* Cerebellar brain inhibition

The study has been registered in the Netherlands Trial Register (NL7321) on October 8, 2018, has been approved by the local medical ethics committee (CMO region Arnhem-Nijmegen), and will be conducted in accordance with the Declaration of Helsinki.

### Objectives

The primary objective of our study is to examine whether a two-week intervention with cerebellar anodal tDCS could lessen ataxia severity in SCA3 patients compared to sham stimulation. The secondary objectives are to investigate if a two-week treatment with cerebellar anodal tDCS (1) diminishes various (cerebellar) non-motor symptoms, (2) enhances the ability of SCA3 patients to acquire conditioned responses in a delay eyeblink classical conditioning paradigm, and (3) restores cerebellar brain inhibition pathways compared to sham stimulation. We will (4) also determine if a single session of tDCS has a transient beneficial effect on ataxia severity.

### Patient population and eligibility criteria

In order to be eligible to participate in this study, a subject must meet all of the following inclusion criteria:

- Age 16 years or older.
- Proven mutation in the *ATXN3* gene.
- SARA score between 3 and 20 at a recent (pre-study) visit. The SARA score reflects ataxia severity and ranges from 0 (no ataxia) to 40 (very severe ataxia) [32]. We previously distinguished between the asymptomatic, preclinical, and ataxic stage of SCAs and proposed definitions for each of these [33]. As about 20% of healthy controls in the SARA validation study were found to have positive ratings in at least one item, we defined clinically manifest ataxia as SARA ≥ 3 and this value will therefore be used as lower cut-off in the current trial. Further analysis of the results of the Benussi et al. study suggested that improvements following cerebellar anodal tDCS were greater in clinically less affected patients [29]. This indicates that a window of opportunity exists, probably related to the fact that in later stages of the disease there is severe neuronal loss and thus an absence of target cells to channel the tDCS effects. In this regard, Rubinsztein and Orr even speculate about a point of no return when a certain threshold of neurodegeneration has been exceeded and beyond which introduction of disease-modifying treatments may be futile [34]. In the current study, we therefore deliberately do not include SCA3 patients who are already wheelchair-bound but rather aim for mildly to moderately affected individuals, using an arbitrary SARA upper cut-off value of 20.

Exclusion criteria are:

- Epilepsy.
- History of brain surgery.
- Metallic implants in or near the skull.
- Presence of a pacemaker.
- Significant comorbidities that interfere with activities of daily life.
- (Suspicion of) pregnancy.
- Severe skin disease affecting the location where the tDCS electrodes will be placed.

Similar to the riluzole trial [16], we deliberately chose not to exclude patients who are receiving physical therapy or who are using centrally acting neurological medication, as we here plan to study the symptomatic effect of cerebellar tDCS added to any ongoing treatment reflecting current daily practice.

Patients will be mainly recruited from our Radboud University Medical Center ataxia outpatient clinic. If they are interested to participate, they will receive a detailed description of the study by post or e-mail and have at least seven days consideration time. In addition, the Dutch ADCA society will help in the recruitment by placing advertisements on their website and in their newsletter. Before enrollment, patients from other centers who are willing to participate are invited to our hospital for a pre-study visit in which the SARA score is determined.

### Randomization and blinding

Participants fulfilling the abovementioned criteria will be randomly assigned in a 1:1 ratio to either cerebellar anodal tDCS or sham cerebellar tDCS using online software provided by Castor EDC. A protocol of permuted block randomization with randomly selected variable block sizes will be applied. Furthermore, in order to ensure an equal distribution of ataxia severity in both groups, the SARA score of a recent (pre-study) visit will be used as a stratification variable. The randomization procedure will be conducted by an independent researcher who subsequently picks one of the 100 five-digit codes from the neuroConn tDCS user manual that belongs to the specific tDCS mode (real or sham stimulation). This person is otherwise not involved in the study. The investigator applying tDCS, other investigators, and patients thus remain unaware of treatment allocation.

### Intervention and study procedures

At the beginning of the first visit, informed consent is obtained from each subject participating in the study after explanation of the aims, methods, benefits, and potential hazards of the study once more. Subjects can withdraw their consent at any time and for any reason without incurring any penalty or withholding of treatment on the part of the investigators.

#### Questionnaires

At various points in time during the study participants are requested to complete a number of questionnaires (Table 1) [5]. The EuroQol 5-Dimension 5-Level (EQ-5D-5L) is an ordinal quality of life scale that contains five questions addressing the domains of mobility, self-care, usual activities, pain / discomfort, and anxiety / depression, each with five answer options. In the final question, subjects rate their overall health on a visual scale from 0 (worst imaginable health status) to 100 (best imaginable health status). The Patient Health Questionnaire-9 (PHQ-9) is a 9-item instrument that is used in the screening, diagnosis, and follow-up of depression. Participants record how often they experienced each of these nine symptoms in the last two weeks, ranging from never (score 0) to nearly every day (score 3), thus resulting in a total score between 0 and 27 points. In the shortened 32-item version of the Profile of Mood States (POMS), 32 adjectives are presented and subjects determine to what extent (0 = not at all to 4 = very well) each of these is applicable to their current state. In this way, mean scores on five different mood domains can be evaluated, namely depression, anger, tensor, fatigue, and vigor. The institute for Medical Technology Assessment Medical Consumption Questionnaire (iMCQ) aims to compute the medical costs made by the participants in the last three months due to physical and mental complaints [35]. These costs are calculated by multiplying the price per unit of care and the volume of care for each item. As a possible confounder, the degree of physical exertion will be assessed using parts 1 and 4 of the long version of the International Physical Activity Questionnaire [36]. These address the amount of moderate and vigorous exercise and time spent walking in the last seven days at work (part 1), if any, and during recreation, sport, and leisure time (part 4), and will be expressed in Metabolic Equivalent of Task (MET)-minutes per week. The number of MET-minutes per week is computed by multiplying the energy requirements of the type of activity – 3.3 for walking, 4.0 for moderate exercise, and 8.0 for vigorous physical activity – with the amount of minutes per day and the number of days per week on which that activity is conducted. The total work MET-minutes and leisure-time MET-minutes per week will be summed. Finally, part II of the Friedreich Ataxia Rating Scale (FARS) will be used to evaluate activities of daily living. This scale includes nine items and ranges from 0 (no assistance needed) to 36 (fully dependent).

#### Cognitive and affective symptoms

In the last decades it has become increasingly clear that the cerebellum is not only involved in motor tasks, but also plays an important role in various cognitive functions, predominantly affect regulation, executive functioning, spatial cognition, and linguistic processing. Deficits in these domains in patients with focal cerebellar lesions or degenerative cerebellar disorders are collectively referred to as the cerebellar cognitive affective syndrome (CCAS) [7, 11, 12, 37, 38]. Recently, Hoche et al. selected the most sensitive tests to screen for cerebellar cognitive and affective dysfunction out of a comprehensive standardized neuropsychological examination and validated this 10-item bedside screening battery test, called the CCAS/Schmahmann syndrome scale, in a new cohort of patients with cerebellar diseases [39]. In addition to a total raw score (0 to 120 points), the authors provided cut-offs for each item, which determine whether a participant passed or failed this test. Possible, probable, and definite CCAS were subsequently defined as a failure at 1, 2, or ≥ 3 items, respectively. In the current study, we will apply this CCAS/Schmahmann syndrome scale and examine the influence of cerebellar tDCS on both the total raw score and the number of failed tests.

#### Neurological examination and quantitative motor tests

A standardized neurological examination will be conducted and includes an assessment of ataxia severity (SARA), which will be videotaped and rated by two highly experienced investigators who are blind to randomization status and point in time. The Inventory of Non-Ataxia Signs (INAS), a clinical assessment instrument containing 30 items, will be used to determine the amount of extracerebellar involvement and results in an INAS count ranging from 0 (no non-ataxia signs) to 16 (very severe extracerebellar signs) [40]. More quantitative motor tests include two trials of the 8MWT, two trials of the 9HPT for each hand, and two trials of the PATA repetition rate task, which provide measures of gait speed, upper limb dexterity, and articulation speed, respectively [41]. In the former, the time will be recorded that it takes for a participant to walk a distance of 8 m as quickly as possible but safely. In the 9HPT, subjects are asked to fill the nine holes of the peg hole board with pegs one at a time as quickly as possible and then, without pausing, to remove them again one at a time. Lastly, in the PATA repetition rate task subjects repeat the “PATA” phrase as quickly as possible for ten seconds. The number of PATA repeats will then be counted.

#### Transcranial magnetic stimulation

The inhibitory influence of the cerebellum on the contralateral primary motor cortex (M1), a physiological phenomenon called cerebellar brain inhibition (CBI), can be evaluated using a dual-coil TMS paradigm. CBI can be demonstrated by a reduction of the motor evoked potential (MEP) amplitude when a test stimulus over M1 is preceded by a conditioning stimulus administered over the contralateral cerebellar hemisphere with an interval of 5 to 7 ms [42]. In patients with cerebellar ataxia due to lesions in the cerebellar efferent pathway, CBI is either significantly reduced or absent [43–45].

The CBI paradigm will be applied at T0 and T1. Conditioning stimuli will be delivered to the posterior and superior lobules of the right cerebellar hemisphere at a point 1 cm below and 3 cm lateral to the inion on the line joining the external auditory meatus. Although both double-cone coils and figure-of-eight coils have been previously utilized for this purpose, a recent systematic review concluded that the majority of CBI studies argued for the former [46]. Because the double-cone coil generates a larger electromagnetic field as compared to the conventional figure-of-eight coils and is therefore able to reach deeper target tissues, we also decided to use this coil (Magstim 110 mm) in the administration of cerebellar conditioning stimuli. Indeed, Hardwick and colleagues could not elicit CBI using the figure-of-eight coil in healthy young individuals, even at intensities of 65–80% maximum stimulator output, whereas CBI was readily present at all intensities when using the double-cone coil [47]. The coil will be oriented such that an upward brain current is achieved. A Magstim 70 mm figure-of-eight coil will be used to excite the hand representation of the left primary motor cortex with the handle pointed 45° posterolaterally to the midsagittal plane. MEPs are registered from the first dorsal interosseus muscle of the right hand through Kendall H69P surface electrodes in a belly-tendon montage.

After localizing and marking the motor hotspot on the tight-fitting cap worn by the participant (in order to guarantee a constant optimal coil position throughout the experiment), the resting motor threshold (rMT) will be assessed. The latter is defined as the lowest stimulator output to elicit MEPs (≥ 50 μV or visible by contraction) in five out of ten trials during muscle relaxation. The intensity of the cerebellar conditioning stimuli is set at 90% rMT of the contralateral M1 [29, 31, 48–51], while the intensity of motor cortex test pulses will be adjusted to produce MEPs with peak-to-peak amplitudes of 0.5–1.0 mV. Although it is true that the studies that delivered cerebellar conditioning stimuli at an intensity of 90% rMT obtained from M1 made use of a figure-of-eight coil, it can be assumed that a similar and more reliable effect can be accomplished by a double-cone coil.

Twenty trials will be conducted for each interstimulus interval (3 ms, 5 ms, 10 ms, and test pulse only) and presented in a pseudorandomized sequence. The ratio between the mean MEP amplitude after application of the dual-coil paradigm and the test stimulus will be calculated for each interstimulus interval.

#### Delay eyeblink classical conditioning

Delay eyeblink classical conditioning refers to a motor learning paradigm, which is dependent on the structural and functional integrity of cerebellar circuits [52]. In healthy individuals repeated time-locked coupling of an auditory tone (conditioned stimulus, CS) and a supraorbital nerve stimulus or corneal air puff (unconditioned stimulus, US) ultimately leads to the presence of a conditioned eyeblink (conditioned response, CR) prior to the US. Patients with degenerative cerebellar disorders and focal cerebellar lesions have been reported to show significantly diminished acquisition of CRs and no retention when multiple eyeblink conditioning sessions are applied [53–55]. Notably, the acquisition process already seems to be disturbed during the preclinical stage of SCA3 [33, 56].

In this study, we aim to investigate whether diminished acquisition of CRs in SCA3 patients is a static trait, which we postulated before, or whether this process could be enhanced by a two-week treatment with cerebellar anodal tDCS. To this end, we will apply a validated protocol previously described by Teo and colleagues and also used in our own study on preclinical SCA3 mutation carriers [56, 57]. Patients will be comfortably seated in a chair, having their back turned to the computer screen so they cannot see when the CS and US are presented. The US consists of an electrical stimulus (250 V, 200 μs duration) generated by a Digitimer DS7A that stimulates the right supraorbital nerve through a pair of Kendall H69P electrodes. The cathode is placed in the supraorbicular fossa, the anode 2 cm above the cathode. The stimulus level will be determined individually by multiplying the sensory threshold intensity by a factor 7 to 10. Two pairs of Kendall H69P electrodes will be used to record the electromyographic activity from both orbicularis oculi muscles. The active and reference electrodes will be placed just caudal to the lower eyelids and 2–3 cm from the lateral canthi, respectively. The CS consists of a loud auditory tone, presented via binaural headphones, and precedes the US by 400 ms, eventually co-terminating with it.

The conditioning protocol comprises six blocks of eleven trials: trials 1–9 are CS-US-paired, trial 10 is CS-only, and trial 11 is US-only. Trial 10 is inserted to ensure that acquisition of CRs occurs independent of the US. Extinction will be tested for by including a seventh block of eleven CS-only trials. Using this protocol, the percentage and timing of acquired conditioned responses will be assessed as well as the occurrence of extinction.

#### Static posturography

Static posturography will be performed to provide a quantitative, kinetic measure of standing balance performance compared to the more descriptive SARA stance item. To this end, subjects are asked to stand barefoot on a 60 × 40 cm force plate (Motekforce Link), which measures the excursion of the center of pressure (CoP) in the anteroposterior and mediolateral directions at a sampling rate of 1000 Hz. For each participant the “optimal” intermalleolar distance will be determined, defined as the smallest distance between the left and right medial malleolus in steps of 5 cm at which a subject stands comfortably without a tendency to fall. This value will be used throughout the study. Fixed lines are applied to the force plate to facilitate these measurements. Participants are instructed to look straight ahead at a fixed target at the wall with their arms relaxed along their body and stand as quietly as possible during three trials of 30 s each with pauses in between. The investigator will stand next to the subject for safety reasons.

#### Cerebellar transcranial direct current stimulation

Cerebellar tDCS will be delivered by two 7 × 5 cm rubber electrodes, which are connected to a neuroConn constant current stimulator. Similar to the set-up applied by Benussi and colleagues [29], the anode – encased in a dampened sponge envelope – will be placed in the midline 2 cm below the inion in order to reach both cerebellar hemispheres, while the cathode is attached to the skin overlying the right deltoid muscle. The anode and cathode are coated with electroconductive gel and paste, respectively, to maintain impedance levels below 5 kΩ throughout the stimulation. Elastic gauzes and tape are used to ensure fixation of the electrodes.

Following a ramp-up time of 30 s, as recommended previously to ensure proper blinding and reduce side effects [58], in which the current intensity is gradually increased from 0 to 2 mA, a maintenance current of 2 mA will be delivered for 20 min in case of real cerebellar stimulation (0.057 mA/cm^2^). Hereafter, the current is gradually decreased to 0 mA again during a fade-out period of 30 s. In sham sessions, the duration of real stimulation by convention is 40 s (i.e., 1200 / 30), directly preceded and followed by 30 s of ramp-up and ramp-down. The remainder of the time (i.e., 1160 s) consists of continuous impedance control without any stimulation. This is accomplished by the occurrence of a small current pulse every 550 ms (110 μA over 15 ms, peak current duration 3 ms) that enables reliable detection of bad electrode contact or disconnection. Both conditions will thus be indistinguishable with respect to electrode set-up, session duration, and physical sensations. After two weeks of stimulation, patients are asked to which group they think they were allocated.

Participants may experience mild itching, prickling, or burning sensations underneath the electrodes that disappear after a while. Considering the extensive exclusion criteria, the screening procedure, and constant monitoring of the subjects, we do not expect serious adverse events. The risks of tDCS are considered negligible. No serious adverse events have been reported [23, 59] and the similar stimulation protocol used by the Italian group yielded no side effects [29]. In any case, the investigator will make sure that the stimulation is fully tolerable at the beginning as well as throughout the experiment. Measurements will be terminated whenever the participant does not tolerate the measurement or whenever the investigator believes it is not safe to proceed.

### Outcome measures

The various outcome measures are listed in Table 2.

### Statistical analysis

Statistical analyses will be conducted using SPSS and, in general, the significance level is set at *p* < 0.05. Where appropriate, we will use either chi-square tests, t-tests, or two-way ANOVAs with TIME as within-subject factor (number of levels depending on the specific outcome measure, ranging from two to five) and TREATMENT as between-subject factor (anodal tDCS versus sham tDCS). Subsequently, Bonferroni-corrected post-hoc testing is performed to explore significant main effects. Possible correlations with functional scores will be addressed using Spearman’s rank-order correlations.

This study is exploratory in nature. In the previously conducted Benussi et al. trial, the authors included 20 patients with a variety of ataxias and were able to demonstrate significant differences between the stimulation and sham groups in SARA, ICARS, 8MWT, and 9HPT [29]. In the current study, the same sample size of 20 SCA3 patients is chosen (*n* = 10 real stimulation, n = 10 sham stimulation). Based on the data presented in the aforementioned study, a calculation (G*Power 3) revealed a power of 83% to detect significant differences when using SARA as the primary outcome measure (effect size 0.46, α = 0.05, sample size 20).

## Discussion

n this randomized, double-blind, sham-controlled trial the effects of a two-week regimen with daily sessions of cerebellar anodal tDCS will be evaluated for the first time in SCA3 patients. Since patients with less severe ataxia tended to have the largest decrease in coordination deficits in a previous pilot study [29], which likely suggests that the volume of viable cerebellar cortex that can be stimulated plays a crucial role, we will include mildly to moderately affected individuals. Although it is the main aim of this trial to investigate the effects of cerebellar tDCS on ataxia severity, a variety of other outcome measures will be used that reflect the spectrum of deficits observed in patients with cerebellar diseases such as SCA3. In fact, ours is the first study in patients with ataxia that also examines the effects of cerebellar tDCS on non-motor parameters. Relating to the clinically-oriented nature of the study, the outcome measures are mainly administered at the outpatient clinic. In addition, a paired-pulse TMS paradigm will be applied to investigate whether the physiological phenomenon of cerebellar brain inhibition – known to be reduced or absent in patients with lesions in the cerebellar efferent pathway – returns after repeated tDCS sessions.

While the focus will be on the immediate post-treatment (T1) versus baseline differences, we plan to have a follow-up of up to one year. It is very important to determine the exact time curve of the effect (if any) in order to establish the repeat frequency of the intervention to design implementation protocols. Furthermore, we will investigate whether transient effects on motor outcomes are already elicited by a single session of tDCS. In this respect, the SARA score and performances on the 8MWT, 9HPT, PATA repetition rate, and static posturography at T0 will all be completed within 30 to 45 min after the end of the cerebellar tDCS, which falls well within the estimated time window of after-effects of a single tDCS bout [60].

Notwithstanding the exponentially increasing use of cerebellar tDCS in both healthy individuals and (the promising results in) patients with various neurological disorders, its underlying mechanism of action has not been entirely clarified. Unlike TMS, which makes use of the principle of electromagnetic induction to directly elicit neuronal depolarization, the weak currents in anodal tDCS are thought to modulate the excitability of the neuronal membrane potential rather than instantaneously evoke action potentials. In this regard, anodal stimulation leads to a shift in the membrane potential towards depolarization, conditional on the degree of alignment between a neuronal membrane and the electric field, while the opposite is true for cathodal stimulation [58]. The situation for cerebellar tDCS, however, is more difficult to predict than with cerebral stimulation given the specific cytoarchitecture and complex gyration pattern of the former. As stressed previously, repeated sessions of anodal tDCS may induce long-lasting (plasticity) effects, which might result from a change in ionic gradients in the extracellular space, augmentation of synapses, modulation of certain receptors and neurotransmitters, redistribution of membrane proteins, induction of specific cellular processes, and/or increased cerebellar perfusion [23, 58].

If we can demonstrate that cerebellar stimulation via anodal tDCS improves ataxia and perhaps non-motor features in SCA3 patients, this would mean that there is a readily available, safe, and affordable tool for its management in the neurorehabilitation setting. Furthermore, positive effects will stimulate further research into this intervention in other SCAs and heredodegenerative ataxias for which effective treatment options are currently lacking. Finally, the observation that extended, remotely supervised at-home tDCS treatment has proven feasible in cerebellar ataxia may facilitate future clinical implementation once the efficacy of this technique has been established on a larger scale [61].

## Data Availability

Data sharing is not applicable to this article as no datasets were generated or analysed (study protocol).

## Abbreviations

8MWT: 8 m walk test
9HPT: 9-hole peg test
ADCA: autosomal dominant cerebellar ataxia
ARCA: autosomal recessive cerebellar ataxia
CBI: cerebellar brain inhibition
CCAS: Cerebellar Cognitive Affective Syndrome
CoP: Center of Pressure
CR: Conditioned response
CS: Conditioned stimulus
EQ-5D-5L: EuroQol 5-Dimension 5-Level
FARS: Friedreich Ataxia Rating Scale
ICARS: International Cooperative Ataxia Rating Scale
iMCQ: Institute for Medical Technology Assessment Medical Consumption Questionnaire
INAS: Inventory of Non-Ataxia Signs
IPAQ: International Physical Activity Questionnaire
MEP: Motor evoked potential
MET: Metabolic Equivalent Task
PHQ-9: Patient Health Questionnaire-9
POMS: Profile of Mood States
SARA: Scale for the Assessment and Rating of Ataxia
SCA: Spinocerebellar ataxia
tDCS: Transcranial direct current stimulation
TMS: Transcranial magnetic stimulation
US: Unconditioned stimulus

## References

[1] Silveira I, Miranda C, Guimaraes L, Moreira MC, Alonso I, Mendonca P, Ferro A, Pinto-Basto J, Coelho J, Ferreirinha F (2002). Trinucleotide repeats in 202 families with ataxia: a small expanded (CAG) n allele at the SCA17 locus. Arch Neurol.

[2] Tang B, Liu C, Shen L, Dai H, Pan Q, Jing L, Ouyang S, Xia J (2000). Frequency of SCA1, SCA2, SCA3/MJD, SCA6, SCA7, and DRPLA CAG trinucleotide repeat expansion in patients with hereditary spinocerebellar ataxia from Chinese kindreds. Arch Neurol.

[3] van de Warrenburg BP, Sinke RJ, Verschuuren-Bemelmans CC, Scheffer H, Brunt ER, Ippel PF, Maat-Kievit JA, Dooijes D, Notermans NC, Lindhout D (2002). Spinocerebellar ataxias in the Netherlands: prevalence and age at onset variance analysis. Neurology.

[4] Sanchez-Lopez CR, Perestelo-Perez L, Escobar A, Lopez-Bastida J, Serrano-Aguilar P (2017). Health-related quality of life in patients with spinocerebellar ataxia. Neurologia.

[5] Schmitz-Hubsch T, Coudert M, Giunti P, Globas C, Baliko L, Fancellu R, Mariotti C, Filla A, Rakowicz M, Charles P (2010). Self-rated health status in spinocerebellar ataxia--results from a European multicenter study. Mov Disord.

[6] Braga-Neto P, Pedroso JL, Alessi H, Dutra LA, Felicio AC, Minett T, Weisman P, Santos-Galduroz RF, Bertolucci PH, Gabbai AA (2012). Cerebellar cognitive affective syndrome in Machado Joseph disease: core clinical features. Cerebellum.

[7] Giocondo F, Curcio G (2018). Spinocerebellar ataxia: a critical review of cognitive and socio-cognitive deficits. Int J Neurosci.

[8] Kawai Y, Takeda A, Abe Y, Washimi Y, Tanaka F, Sobue G (2004). Cognitive impairments in Machado-Joseph disease. Arch Neurol.

[9] Lindsay E, Storey E. Cognitive changes in the spinocerebellar ataxias due to expanded Polyglutamine tracts: a survey of the literature. Brain Sci. 2017;7(7). 10.3390/brainsci7070083 PMC5532596 28708110

[10] Roeske S, Filla I, Heim S, Amunts K, Helmstaedter C, Wullner U, Wagner M, Klockgether T, Minnerop M (2013). Progressive cognitive dysfunction in spinocerebellar ataxia type 3. Mov Disord.

[11] Schmahmann JD, Sherman JC (1998). The cerebellar cognitive affective syndrome. Brain.

[12] Stoodley CJ, Schmahmann JD (2010). Evidence for topographic organization in the cerebellum of motor control versus cognitive and affective processing. Cortex.

[13] Zawacki TM, Grace J, Friedman JH, Sudarsky L (2002). Executive and emotional dysfunction in Machado-Joseph disease. Mov Disord.

[14] van de Warrenburg BP, van Gaalen J, Boesch S, Burgunder JM, Durr A, Giunti P, Klockgether T, Mariotti C, Pandolfo M, Riess O (2014). EFNS/ENS consensus on the diagnosis and management of chronic ataxias in adulthood. Eur J Neurol.

[15] Zesiewicz Theresa A., Wilmot George, Kuo Sheng-Han, Perlman Susan, Greenstein Patricia E., Ying Sarah H., Ashizawa Tetsuo, Subramony S.H., Schmahmann Jeremy D., Figueroa K.P., Mizusawa Hidehiro, Schöls Ludger, Shaw Jessica D., Dubinsky Richard M., Armstrong Melissa J., Gronseth Gary S., Sullivan Kelly L. (2018). Comprehensive systematic review summary: Treatment of cerebellar motor dysfunction and ataxia. Neurology.

[16] Romano S, Coarelli G, Marcotulli C, Leonardi L, Piccolo F, Spadaro M, Frontali M, Ferraldeschi M, Vulpiani MC, Ponzelli F (2015). Riluzole in patients with hereditary cerebellar ataxia: a randomised, double-blind, placebo-controlled trial. Lancet Neurol.

[17] Fonteyn EM, Keus SH, Verstappen CC, van de Warrenburg BP (2013). Physiotherapy in degenerative cerebellar ataxias: utilisation, patient satisfaction, and professional expertise. Cerebellum.

[18] Galea JM, Jayaram G, Ajagbe L, Celnik P (2009). Modulation of cerebellar excitability by polarity-specific noninvasive direct current stimulation. J Neurosci.

[19] Oldrati V, Schutter D (2018). Targeting the human cerebellum with transcranial direct current stimulation to modulate behavior: a meta-analysis. Cerebellum.

[20] Parazzini M, Rossi E, Ferrucci R, Liorni I, Priori A, Ravazzani P (2014). Modelling the electric field and the current density generated by cerebellar transcranial DC stimulation in humans. Clin Neurophysiol.

[21] Rampersad SM, Janssen AM, Lucka F, Aydin U, Lanfer B, Lew S, Wolters CH, Stegeman DF, Oostendorp TF (2014). Simulating transcranial direct current stimulation with a detailed anisotropic human head model. IEEE Trans Neural Syst Rehabil Eng.

[22] Ferrucci R, Priori A (2014). Transcranial cerebellar direct current stimulation (tcDCS): motor control, cognition, learning and emotions. Neuroimage.

[23] Grimaldi G, Argyropoulos GP, Bastian A, Cortes M, Davis NJ, Edwards DJ, Ferrucci R, Fregni F, Galea JM, Hamada M (2016). Cerebellar transcranial direct current stimulation (ctDCS): a novel approach to understanding cerebellar function in health and disease. Neuroscientist.

[24] Ferrucci R, Bocci T, Cortese F, Ruggiero F, Priori A (2016). Cerebellar transcranial direct current stimulation in neurological disease. Cerebellum Ataxias.

[25] Franca C, de Andrade DC, Teixeira MJ, Galhardoni R, Silva V, Barbosa ER, Cury RG (2018). Effects of cerebellar neuromodulation in movement disorders: a systematic review. Brain Stimul.

[26] Bodranghien F, Oulad Ben Taib N, Van Maldergem L, Manto M (2017). A postural tremor highly responsive to transcranial Cerebello-cerebral DCS in ARCA3. Front Neurol.

[27] Grimaldi G, Oulad Ben Taib N, Manto M, Bodranghien F (2014). Marked reduction of cerebellar deficits in upper limbs following transcranial cerebello-cerebral DC stimulation: tremor reduction and re-programming of the timing of antagonist commands. Front Syst Neurosci.

[28] Benussi A, Koch G, Cotelli M, Padovani A, Borroni B (2015). Cerebellar transcranial direct current stimulation in patients with ataxia: a double-blind, randomized, sham-controlled study. Mov Disord.

[29] Benussi A, Dell'Era V, Cotelli MS, Turla M, Casali C, Padovani A, Borroni B (2017). Long term clinical and neurophysiological effects of cerebellar transcranial direct current stimulation in patients with neurodegenerative ataxia. Brain Stimul.

[30] Jacobi H, du Montcel ST, Bauer P, Giunti P, Cook A, Labrum R, Parkinson MH, Durr A, Brice A, Charles P (2015). Long-term disease progression in spinocerebellar ataxia types 1, 2, 3, and 6: a longitudinal cohort study. Lancet Neurol.

[31] Benussi A, Dell'Era V, Cantoni V, Bonetta E, Grasso R, Manenti R, Cotelli M, Padovani A, Borroni B (2018). Cerebello-spinal tDCS in ataxia: a randomized, double-blind, sham-controlled, crossover trial. Neurology.

[32] Schmitz-Hubsch T, du Montcel ST, Baliko L, Berciano J, Boesch S, Depondt C, Giunti P, Globas C, Infante J, Kang JS (2006). Scale for the assessment and rating of ataxia: development of a new clinical scale. Neurology.

[33] Maas RP, van Gaalen J, Klockgether T, van de Warrenburg BP (2015). The preclinical stage of spinocerebellar ataxias. Neurology.

[34] Rubinsztein DC, Orr HT (2016). Diminishing return for mechanistic therapeutics with neurodegenerative disease duration?: there may be a point in the course of a neurodegenerative condition where therapeutics targeting disease-causing mechanisms are futile. Bioessays.

[35] http://www.imta.nl (Bouwmans C, Hakkaart-van Roijen L, Koopmanschap M, Krol M, Severens H, Brouwer W: Handleiding *i*MTA Medical Cost Questionnaire (*i*MCQ)**.** Rotterdam*:* iMTA, Erasmus Universiteit Rotterdam*,* 2013).

[36] Craig CL, Marshall AL, Sjostrom M, Bauman AE, Booth ML, Ainsworth BE, Pratt M, Ekelund U, Yngve A, Sallis JF (2003). International physical activity questionnaire: 12-country reliability and validity. Med Sci Sports Exerc.

[37] Buckner RL (2013). The cerebellum and cognitive function: 25 years of insight from anatomy and neuroimaging. Neuron.

[38] Schmahmann JD (1998). Dysmetria of thought: clinical consequences of cerebellar dysfunction on cognition and affect. Trends Cogn Sci.

[39] Hoche F, Guell X, Vangel MG, Sherman JC, Schmahmann JD (2018). The cerebellar cognitive affective/Schmahmann syndrome scale. Brain.

[40] Jacobi H, Rakowicz M, Rola R, Fancellu R, Mariotti C, Charles P, Durr A, Kuper M, Timmann D, Linnemann C (2013). Inventory of non-Ataxia signs (INAS): validation of a new clinical assessment instrument. Cerebellum.

[41] Schmitz-Hubsch T, Giunti P, Stephenson DA, Globas C, Baliko L, Sacca F, Mariotti C, Rakowicz M, Szymanski S, Infante J (2008). SCA functional index: a useful compound performance measure for spinocerebellar ataxia. Neurology.

[42] Ugawa Y, Uesaka Y, Terao Y, Hanajima R, Kanazawa I (1995). Magnetic stimulation over the cerebellum in humans. Ann Neurol.

[43] Iwata NK, Ugawa Y (2005). The effects of cerebellar stimulation on the motor cortical excitability in neurological disorders: a review. Cerebellum.

[44] Kikuchi S, Mochizuki H, Moriya A, Nakatani-Enomoto S, Nakamura K, Hanajima R, Ugawa Y (2012). Ataxic hemiparesis: neurophysiological analysis by cerebellar transcranial magnetic stimulation. Cerebellum.

[45] Ugawa Y, Terao Y, Hanajima R, Sakai K, Furubayashi T, Machii K, Kanazawa I (1997). Magnetic stimulation over the cerebellum in patients with ataxia. Electroencephalogr Clin Neurophysiol.

[46] Fernandez L, Major BP, Teo WP, Byrne LK, Enticott PG (2018). Assessing cerebellar brain inhibition (CBI) via transcranial magnetic stimulation (TMS): a systematic review. Neurosci Biobehav Rev.

[47] Hardwick RM, Lesage E, Miall RC (2014). Cerebellar transcranial magnetic stimulation: the role of coil geometry and tissue depth. Brain Stimul.

[48] Brusa L, Ponzo V, Mastropasqua C, Picazio S, Bonni S, Di Lorenzo F, Iani C, Stefani A, Stanzione P, Caltagirone C (2014). Theta burst stimulation modulates cerebellar-cortical connectivity in patients with progressive supranuclear palsy. Brain Stimul.

[49] Carrillo F, Palomar FJ, Conde V, Diaz-Corrales FJ, Porcacchia P, Fernandez-Del-Olmo M, Koch G, Mir P (2013). Study of cerebello-thalamocortical pathway by transcranial magnetic stimulation in Parkinson's disease. Brain Stimul.

[50] Koch G, Porcacchia P, Ponzo V, Carrillo F, Caceres-Redondo MT, Brusa L, Desiato MT, Arciprete F, Di Lorenzo F, Pisani A (2014). Effects of two weeks of cerebellar theta burst stimulation in cervical dystonia patients. Brain Stimul.

[51] Popa T, Russo M, Meunier S (2010). Long-lasting inhibition of cerebellar output. Brain Stimul.

[52] Gerwig M, Kolb FP, Timmann D (2007). The involvement of the human cerebellum in eyeblink conditioning. Cerebellum.

[53] Gerwig M, Guberina H, Esser AC, Siebler M, Schoch B, Frings M, Kolb FP, Aurich V, Beck A, Forsting M (2010). Evaluation of multiple-session delay eyeblink conditioning comparing patients with focal cerebellar lesions and cerebellar degeneration. Behav Brain Res.

[54] Gerwig M, Hajjar K, Dimitrova A, Maschke M, Kolb FP, Frings M, Thilmann AF, Forsting M, Diener HC, Timmann D (2005). Timing of conditioned eyeblink responses is impaired in cerebellar patients. J Neurosci.

[55] Timmann D, Gerwig M, Frings M, Maschke M, Kolb FP (2005). Eyeblink conditioning in patients with hereditary ataxia: a one-year follow-up study. Exp Brain Res.

[56] van Gaalen J, Maas R, Ippel EF, Elting MW, van Spaendonck-Zwarts KY, Vermeer S, Verschuuren-Bemelmans C, Timmann D, van de Warrenburg BP. Abnormal eyeblink conditioning is an early marker of cerebellar dysfunction in preclinical SCA3 mutation carriers. Exp Brain Res. 2019;237(2):427–33. 10.1007/s00221-018-5424-y PMC6373441 30430184

[57] Teo JT, van de Warrenburg BP, Schneider SA, Rothwell JC, Bhatia KP (2009). Neurophysiological evidence for cerebellar dysfunction in primary focal dystonia. J Neurol Neurosurg Psychiatry.

[58] van Dun K, Bodranghien FC, Marien P, Manto MU (2016). tDCS of the cerebellum: where do we stand in 2016? Technical issues and critical review of the literature. Front Hum Neurosci.

[59] Nitsche MA, Cohen LG, Wassermann EM, Priori A, Lang N, Antal A, Paulus W, Hummel F, Boggio PS, Fregni F (2008). Transcranial direct current stimulation: state of the art 2008. Brain Stimul.

[60] Brunoni AR, Nitsche MA, Bolognini N, Bikson M, Wagner T, Merabet L, Edwards DJ, Valero-Cabre A, Rotenberg A, Pascual-Leone A (2012). Clinical research with transcranial direct current stimulation (tDCS): challenges and future directions. Brain Stimul.

[61] Pilloni G, Shaw M, Feinberg C, Clayton A, Palmeri M, Datta A, Charvet LE (2019). Long term at-home treatment with transcranial direct current stimulation (tDCS) improves symptoms of cerebellar ataxia: a case report. J Neuroeng Rehabil.

