# Correction to: Cerebellar transcranial direct current stimulation in spinocerebellar ataxia type 3 (SCA3-tDCS): rationale and protocol of a randomized, double-blind, sham-controlled study

**DOI:** 10.1186/s12883-021-02278-6

**Published:** 2021-06-29

**Authors:** Roderick P. P. W. M. Maas, Ivan Toni, Jonne Doorduin, Thomas Klockgether, Dennis J. L. G. Schutter, Bart P. C. van de Warrenburg

**Affiliations:** 1 Department of Neurology, Donders Institute for Brain, Cognition, and Behaviour, Radboud University Medical Center, Reinier Postlaan 4, 6525 GC Nijmegen, The Netherlands; 2 Donders Institute for Brain, Cognition, and Behaviour, Radboud University, Nijmegen, The Netherlands; 3 Department of Neurology, University of Bonn, Bonn, Germany; 4 German Center for Neurodegenerative Diseases (DZNE), Bonn, Germany

**Correction to: BMC Neurol 19, 149 (2019)**

**https://doi.org/10.1186/s12883-019-1379-2**

Following publication of the original article [1], the authors noticed an error in the power calculation. The reported power of 0.83 was based on an effect size of 0.46 rather than a partial *η*^*2*^ value of 0.46. Using a partial *η*^*2*^ value of 0.46, which corresponds to an effect size *f* of 0.92, and five repeated measurements in 20 participants would yield a power of 0.999. The original sentence “Based on the data presented in the aforementioned study, a calculation (G*Power 3) revealed a power of 83% to detect significant differences when using SARA as the primary outcome measure (effect size 0.46, α = 0.05, sample size 20)” is therefore replaced as follows: “Based on the reported partial *η*^*2*^ value of 0.46 in the aforementioned study, which corresponds to an effect size *f* of 0.92, a sample size of 20 participants who each have five measurements would yield an estimated power of 0.999 (G*Power 3.1) to detect significant differences when using SARA as the primary outcome measure (α = 0.05).”

The original article has been corrected.
